# Identification of tumor antigens and immune subtypes of cholangiocarcinoma for mRNA vaccine development

**DOI:** 10.1186/s12943-021-01342-6

**Published:** 2021-03-08

**Authors:** Xing Huang, Tianyu Tang, Gang Zhang, Tingbo Liang

**Affiliations:** 1 Department of Hepatobiliary and Pancreatic Surgery, the First Affiliated Hospital, School of Medicine, Zhejiang University, 79 Qingchun Road, Hangzhou, 310003 Zhejiang China; 2 Zhejiang Provincial Key Laboratory of Pancreatic Disease, the First Affiliated Hospital, School of Medicine, Zhejiang University, Hangzhou, 310003 Zhejiang China; 3 Innovation Center for the Study of Pancreatic Diseases, Hangzhou, 310003 Zhejiang Province China; 4 Cancer Center, Zhejiang University, Hangzhou, 310058 Zhejiang China; 5 Research Center for Healthcare Data Science, Zhejiang Lab, Hangzhou, 310003 Zhejiang China

**Keywords:** mRNA vaccine, Cholangiocarcinoma, Tumor antigens, Immune subtypes, Immune landscape

## Abstract

**Background:**

The mRNA-based cancer vaccine has been considered a promising strategy and the next hotspot in cancer immunotherapy. However, its application on cholangiocarcinoma remains largely uncharacterized. This study aimed to identify potential antigens of cholangiocarcinoma for development of anti-cholangiocarcinoma mRNA vaccine, and determine immune subtypes of cholangiocarcinoma for selection of suitable patients from an extremely heterogeneous population.

**Methods:**

Gene expression profiles and corresponding clinical information were collected from GEO and TCGA, respectively. cBioPortal was used to visualize and compare genetic alterations. GEPIA2 was used to calculate the prognostic index of the selected antigens. TIMER was used to visualize the correlation between the infiltration of antigen-presenting cells and the expression of the identified antigens. Consensus clustering analysis was performed to identify the immune subtypes. Graph learning-based dimensionality reduction analysis was conducted to visualize the immune landscape of cholangiocarcinoma.

**Results:**

Three tumor antigens, such as CD247, FCGR1A, and TRRAP, correlated with superior prognoses and infiltration of antigen-presenting cells were identified in cholangiocarcinoma. Cholangiocarcinoma patients were stratified into two immune subtypes characterized by differential molecular, cellular and clinical features. Patients with the IS1 tumor had immune “hot” and immunosuppressive phenotype, whereas those with the IS2 tumor had immune “cold” phenotype. Interestingly, patients with the IS2 tumor had a superior survival than those with the IS1 tumor. Furthermore, distinct expression of immune checkpoints and immunogenic cell death modulators was observed between different immune subtype tumors. Finally, the immune landscape of cholangiocarcinoma revealed immune cell components in individual patient.

**Conclusions:**

CD247, FCGR1A, and TRRAP are potential antigens for mRNA vaccine development against cholangiocarcinoma, specifically for patients with IS2 tumors. Therefore, this study provides a theoretical basis for the anti-cholangiocarcinoma mRNA vaccine and defines suitable patients for vaccination.

## Background

Cholangiocarcinoma (CHOL) is one of the most aggressive and lethal malignancies [1, 2]. At present, surgical resection is the only available treatment to cure CHOL. However, most patients miss the opportunity to be subjected to surgery due to the advanced disease stage at diagnosis, caused by its “silent” clinical characteristics [1, 2]. Besides, the systemic treatment is still limited in these patients at advanced stages. The combination of gemcitabine and cisplatin is the first-line treatment but with a limited response rate and high risk of primary and acquired resistance [1, 3]; and thus the prognosis of these patients is extremely poor, with a median overall survival (OS) of less than one year [3]. Therefore, novel strategies are needed to improve the therapeutic condition of CHOL.

To date, cancer immunotherapy has achieved considerable success in combatting several malignancies [4–6]. Following immune checkpoint inhibitors targeting programmed cell death protein 1 and its ligand 1, the mRNA-cancer vaccine has become increasingly attractive to scientists and oncologists and could be a hotspot in cancer immunotherapy [7, 8]. Actually, mRNA-based therapy was not common before the 2000s due to the instability of mRNA and related excessive inflammation responses. However, technological breakthroughs, including incorporation of modified nucleosides, purification of IVT mRNA, optimization of coding sequences, and development of efficacious delivery material, changed the situation by enabling mRNA optimal form to carry tumor antigens [9, 10]. For instance, mRNA sequence can be easily modified to encode any protein, unlike the traditional peptide vaccine that requires genetic analysis of cancer. This greatly improves the productivity of the vaccine and shortens therapeutic empty window of the patients. Importantly, the half-life of mRNA is adjustable through a delivery system or RNA sequence modification for safety. Moreover, mRNA has no gene integration risk and irrelevant sequence exclusion caused by DNA type, preventing insertional mutagenesis or gene deletion. The self-adjuvant properties of mRNA (e.g., cytokines) increase its in vivo immunogenicity and induce a strong and persistent immune response [7–10]. Preclinical models have demonstrated that the vaccine encoding tumor-specific antigens promotes an anti-tumor immunity and prevents multiple tumors, including melanoma, hepatocellular carcinoma, colorectal cancer, gastrointestinal cancer, and pancreatic adenocarcinoma [7–13].

However, CHOL still lacks an effective mRNA vaccine as the isolation of potent antigens for anti-CHOL mRNA vaccine from hundreds of thousands of mutated candidates is still challenging. Moreover, only a small fraction of CHOL patients might benefit from mRNA vaccine due to tumor heterogeneity and its complex immune microenvironment (TIME) [14–16]. Therefore, patient stratification based on tumor biological subtypes can be used to identify suitable patients for vaccination. Previous CHOL classification was based on certain molecular patterns and mainly focused on tumor cell-intrinsic molecular profile, including gene amplification, copy number alterations, and signaling pathways deregulation [15–17]. However, this traditional method is not sufficient to screen applicable candidates for mRNA vaccine in perspective of immune regulation. In contrast, stratification in light of immune gene expression profile is potentially suitable for identifying patients for mRNA vaccination from an immunologically heterogeneous population.

This study aimed to identify the potential tumor antigens for anti-CHOL mRNA vaccine development. Immunotyping for identifying suitable CHOL patients for vaccination was also investigated. Three tumor antigens correlated with superior prognoses and infiltration of antigen-presenting cells in CHOL were identified, and CHOL patients were stratified into two immune subtypes. The two immune subtypes were associated with differential cellular, molecular and clinical features which were consistent in different cohorts. Our findings might provide valuable information to scientists and oncologists and serve as a reliable reference for further developing and administering cancer vaccines.

## Methods

### Identification of tumor antigens

#### cBioPortal analysis

cBioPortal for Cancer Genomics (cBioPortal, http://www.cbioportal.org, version v3.2.11) is an open-access online tool integrating the raw data from large scale genomic projects including, but not limited to, The Cancer Genome Atlas (TCGA) and the International Cancer Genome Consortium (ICGC) [18]. In this study, cBioPortal was used to visualize the gene alteration of potential antigens against tumors in the TCGA.

#### GEPIA analysis

Gene Expression Profiling Interactive Analysis (GEPIA, http://gepia2.cancer-pku.cn, version 2) is an open-access online tool for the interactive exploration of RNA sequencing data of 9736 tumors and 8587 normal samples from the TCGA and the Genotype-Tissue Expression (GTEx) programs [19]. In this study, GEPIA2 was used to calculate the prognostic index of each selected antigen. The evaluation of the OS and disease-free survival (DFS) of the patients in whom the identified antigens were targeted was performed using the Kaplan-Meier method with a 50% (Median) cutoff for both low and high expression groups. Logrank test (the Mantel-Cox test) was used for hypothesis testing, and a *P*-value < 0.05 was considered statistically significant.

#### TIMER analysis

Tumor Immune Estimation Resource (TIMER, https://cistrome.shinyapps.io/timer/) is a comprehensive resource for the systematical analysis of the immune infiltrates across diverse cancer types [20]. In this study, TIMER was used to visualize the correlation between antigen-presenting cell (APC) infiltration and the expression of the identified potent antigens. The partial Spearman’s correlation was used to perform purity adjustment. Spearman correlation analysis was used to analyze the correlation between the abundance of APCs and the expression of the selected antigens. Statistical significance was set at *P* < 0.05.

### Identification of the immune subtypes

#### Immune-related gene data extraction

A total of 30 CHOL gene expression data and corresponding clinical information were collected from the GEO database (discovery cohort). A total of 45 TGCA database-derived gene expression profile of CHOL were obtained from the UCSC database (validation cohort). A total of 2006 immune-related genes were identified through literature reviewing, including single-cell RNA-seq-derived immune cell-specific genes, co-stimulatory and co-inhibitory molecules, cytokine and cytokine receptors, antigen-presenting, and other immune-related genes.

#### Data preprocessing

The RNA sequence gene expression data of the tumor samples were expressed as reads per kilobase million (RPKM), which was transformed into transcript per million (TPM) to discover the cohorts. A comprehensive set of genes reflecting various immunological processes was collected by mapping the transcription profile using GeneSymbol. Finally, 30 CHOL samples with 1939 immune-related genes were collected. Normal tissues and tumor samples without complete clinical information were excluded to obtain the validation cohort. Genes without expression variance were also excluded. Finally, each patient gene expression was transformed using log2.

#### Identification of immune subtypes and validation

After preprocessing the immune-related gene, the partition around medoids (PAM) algorithm was applied with the “1-Pearson correlation” distance metric, and 500 bootstraps were performed, each comprising 80% of patients of the discovery cohort. Since the discovery cohort was relatively small, patients were clustered into two subtypes. The in-group-proportion (IGP) analysis was performed to quantitatively evaluate the similarity and reproducibility of the identified immune subtypes between two cohorts. The IGP values varied from 0 to 1. A higher IGP for a subtype corresponded to a more reproducible fraction of patients for specific subtypes.

#### Immune-related molecular and cellular features

Biological process enrichment analysis was performed using the “clusterProfiler” package to explore the signaling pathways associated with the immune-related molecular and cellular features. The relationship between the immune subtypes and 56 immune-related molecular and cellular features was assessed [21]. The immune cell composition in the tumor tissue deduced by the CIBERSORT algorithm was analyzed.

#### Gene co-expression network

The R software package WGCNA was used to identify the co-expression modules of the immune-related genes [22]. The hallmark gene sets were downloaded from the Molecular Signatures Database (MSigDB) and GSEA was applied to evaluate the enrichment of these sets. Input genes were ranked in descending order according to the log2FC values. The default settings and 1000 permutations were used to estimate the enrichment significance. Benjamini–Hochberg-adjusted *P*-values less than 0.05 were considered statistically significant.

#### Immune landscape analysis

The graph learning-based dimensionality reduction analysis was performed using the reduceDimension function of the Monocle package with a Gaussian distribution. The maximum number of components was set to 4, and the discriminative dimensionality reduction with trees (DDRTree) was used for dimension reduction. Finally, the function plot_cell_trajectory (package Monocle) was used to visualize the immune landscape, with different colors corresponding to the different immune subtypes identified above.

## Results

### Identification of potential tumor antigens in CHOL

**Fig. 1:**
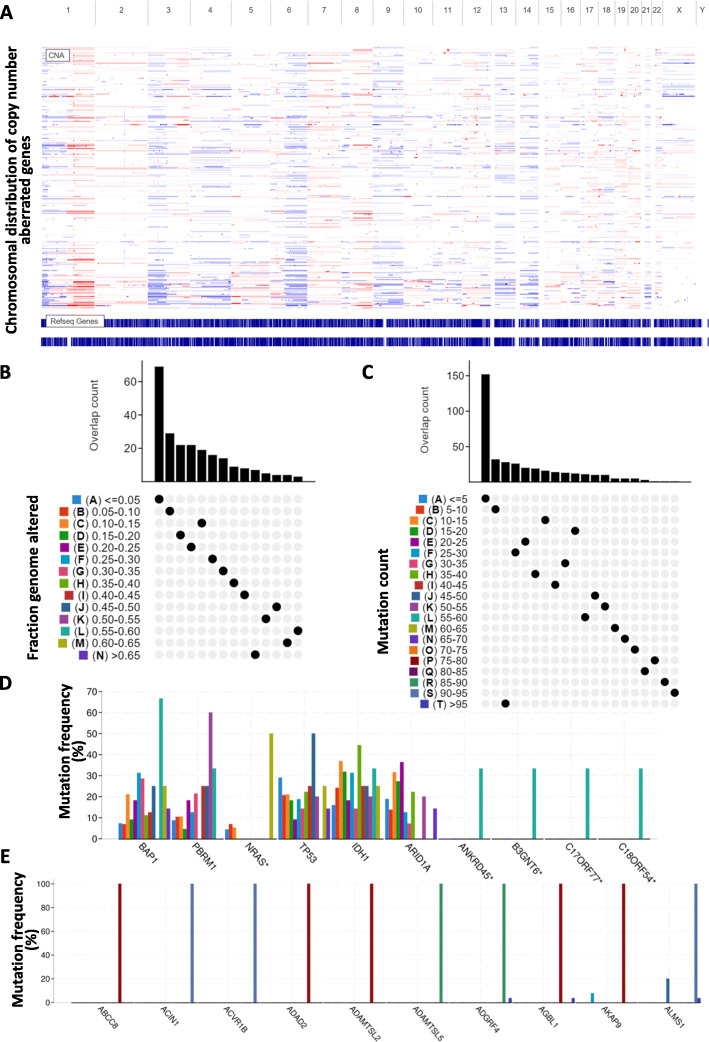
Identification of potential tumor antigens in CHOL. **a** Identification of potential tumor-associated antigens in CHOL. The chromosomal distribution of the aberrant copy number genes in CHOL is shown. **b**-**e** Identification of potential tumor-specific antigens in CHOL. Overlapping mutated genes distributed in the fraction genome altered group (**b**) and mutation count group (**c**) are shown. Genes with the highest frequency in the fraction genome altered groups (**d**) and mutation count groups (**e**) are individually shown

The copy number of aberrated genes were first explored, where 4276 amplified genes that could express the tumor-associated antigens were screened to identify potent CHOL antigens (Fig. 1a). Subsequently, a total of 7283 mutated genes encoding tumor-specific antigens were screened by assessing fraction genome alteration and mutation count in each patient. Most patients showed low fraction genome alteration and mutation count, indicating that CHOL is characterized by low immunogenicity (Fig. 1b and c). Genes with the highest alteration frequency in the fraction genome altered group, including BRCA1 associated protein 1, polybromo 1, tumor protein p53, isocitrate dehydrogenase (NADP(+)) 1, and at-rich interaction domain 1A, were individually displayed (Fig. 1d). Genes with the highest mutation frequency in the mutation count group, including ATP binding cassette subfamily C member 8, apoptotic chromatin condensation inducer 1, activin A receptor type 1B, adenosine deaminase domain containing 2, ADAMTS Like 2, ADAMTS Like 5, adhesion G protein-coupled receptor F4, ATP/GTP binding protein-like 1, A-kinase anchoring protein 9, and ALMS1 centrosome and basal body associated protein, were individually displayed (Fig. 1e). Taken together, 1391 amplified and mutated genes were identified.

### Identification of tumor antigens associated with CHOL prognosis and antigen-presenting cells

**Fig. 2:**
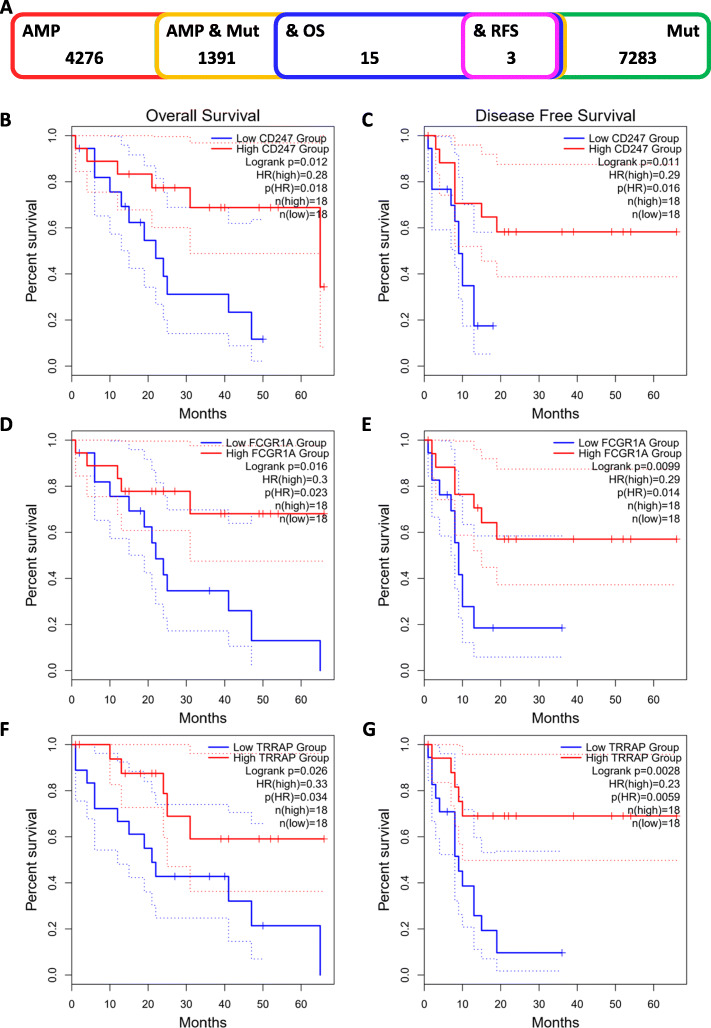
Identification of tumor antigens associated with CHOL prognosis. **a** Narrow-down analysis of potential tumor antigens with both amplified and mutated features (in a total of 1391 candidates), and significant OS and RFS prognosis (in a total of 3 candidates) in CHOL. **b**-**c** Kaplan-Meier OS (**b**) and RFS (**c**) curves comparing the groups with a different CD247 expression in CHOL. **d**-**e** Kaplan-Meier OS (**d**) and RFS (**e**) curves comparing the groups with a different expression of FCGR1A in CHOL. **f**-**g** Kaplan-Meier OS (**f**) and RFS (**g**) curves comparing the groups with a different expression of TRRAP in CHOL

**Fig. 3:**
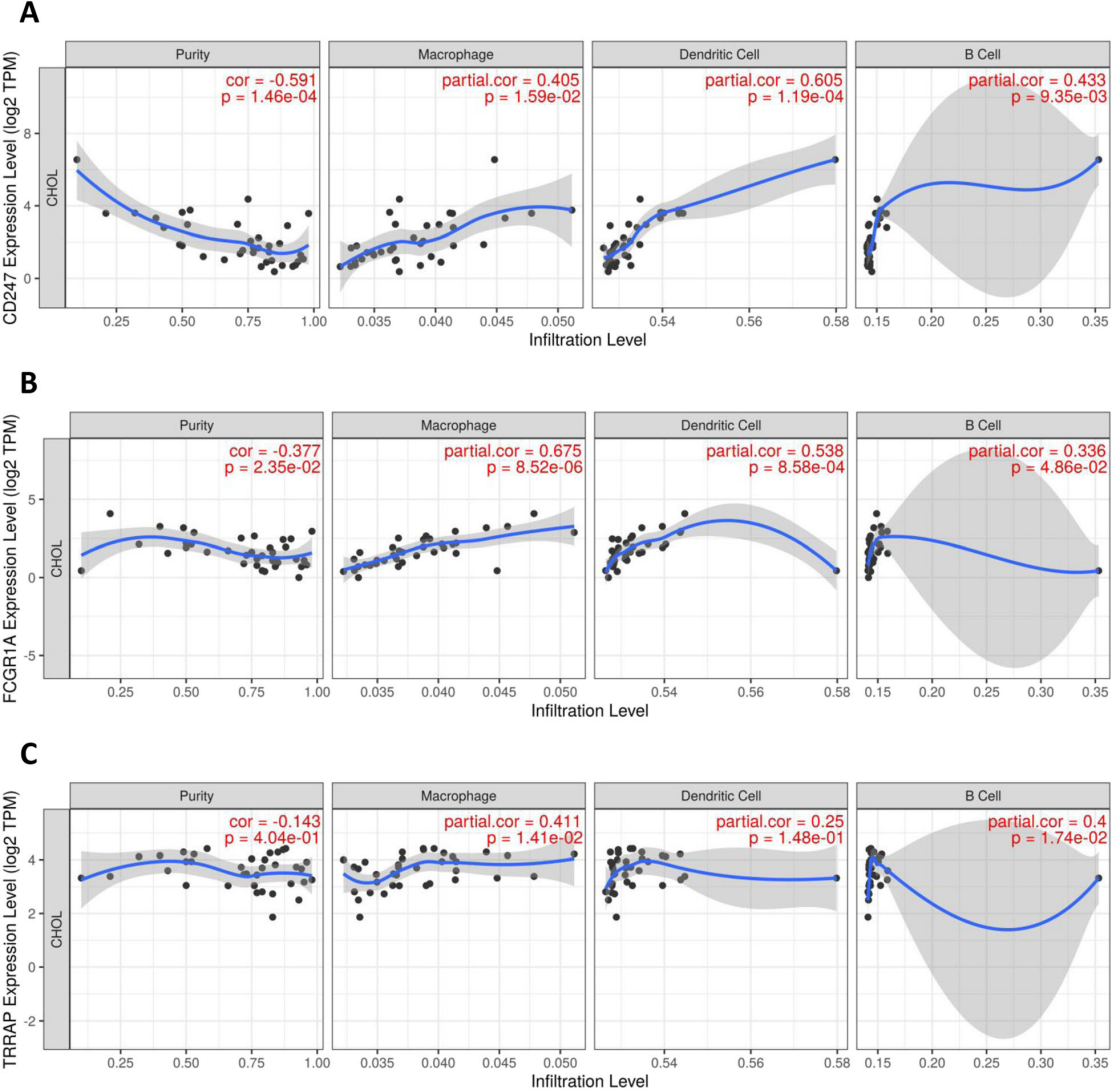
Identification of tumor antigens associated with antigen-presenting cells. **a** Association of CD247 expression with the purity of infiltrating cells and amount of macrophages, dendritic cells, and B cells in CHOL. **b** Association of FCGR1A expression with the purity of infiltrating cells and amount of macrophages, dendritic cells, and B cells in CHOL. **c** Association of TRRAP expression with the purity of infiltrating cells and amount of macrophages, dendritic cells, and B cells in CHOL

The survival relevance of the amplified and mutated genes was analyzed to further screen prognostically relevant antigens that may have immune-stimulatory or inhibitory effects as candidates for mRNA vaccine development. A total of 15 genes closely correlated with the OS of CHOL were identified, where three were related to the RFS (Fig. 2a). The elevated expression of CD247 (Fig. 2b and c), FCGR1A (Fig. 2d and e) and TRRAP (Fig. 2f and g) were associated with superior OS and RFS of CHOL, indicating that the three tumor antigens have potential immune-stimulatory effects. Importantly, the expression levels of CD247 (Fig. 3a) and FCGR1A (Fig. 3b) were positively correlated with the levels of macrophages, Dendritic cells (DCs), and B cells. Although more fluctuant, the higher TRRAP expression was also positively correlated with the levels of macrophages, DCs, and B cells (Fig. 3c). Together, three tumor antigens (CD247, FCGR1A, and TRRAP) were identified as potential candidates for the CHOL-mRNA vaccine with potential immune provocative effects and can be processed and presented by antigen-presenting cells (APCs) to induce a tumor response.

### Identification of potential immune subtypes of CHOL

**Fig. 4:**
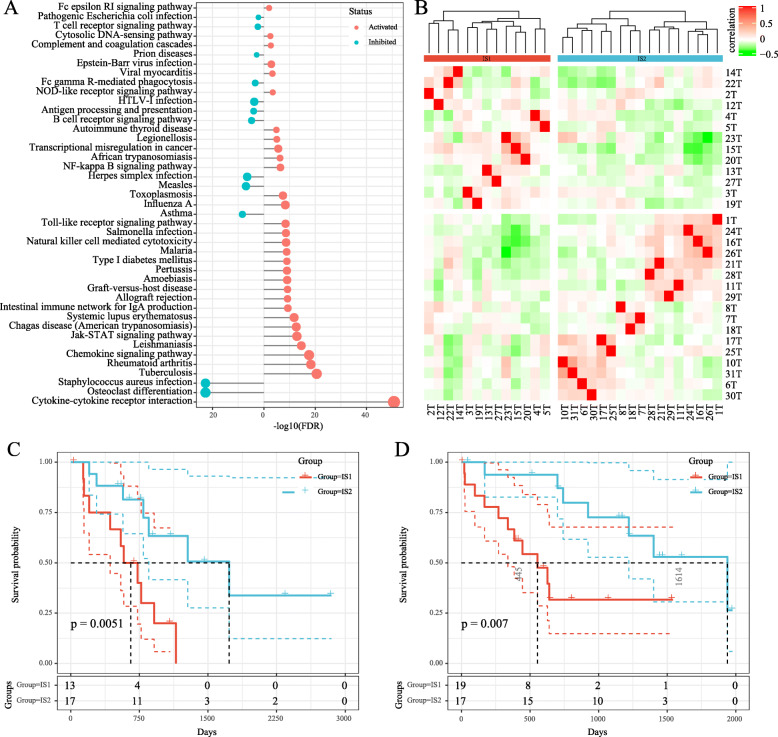
Identification of potential immune subtypes of CHOL. **a** Enrichment analysis of the biological process regulating the immune-related genes. **b** Consensus clustering analysis of CHOL patients based on the immune-related gene expression profile. **c** Survival analysis of the immune subtypes in the GEO cohort. **d** Survival analysis of the immune subtypes in TCGA cohort

A total of 1939 immune gene profiles of CHOL were first extracted and 393 were screened as prognostically related genes to identify suitable patients for vaccination. Further signaling pathway impact analysis (SPIA) suggested that 31 prognosis-related genes enriched signaling pathways were activated (e.g., Natural killer cell-mediated cytotoxicity, cytokine-cytokine receptor interaction), while 12 signaling pathways were inhibited (e.g., T cell receptor signaling pathway, antigen processing and presentation, B cell receptor signaling receptor) (Fig. 4a). Since the activation and inhibition of the signaling pathways were closely associated with the effectiveness of the mRNA vaccine, the prognosis-related gene profiles were further used to construct a consensus cluster. Two immune subtypes (IS1 and IS2) were obtained with the minimum variance within the group and the maximum variance across the groups (Fig. 4b). IS2 had a superior survival probability than IS1 in both GEO and TCGA cohorts (Fig. 4c and d), indicating the reproducibility and stability of the results. Therefore, immunotyping can be a prognostic biomarker, and patients with IS2 tumors could have better prognoses.

### Association of immune subtypes with tumor mutational burden and mutation

**Fig. 5:**
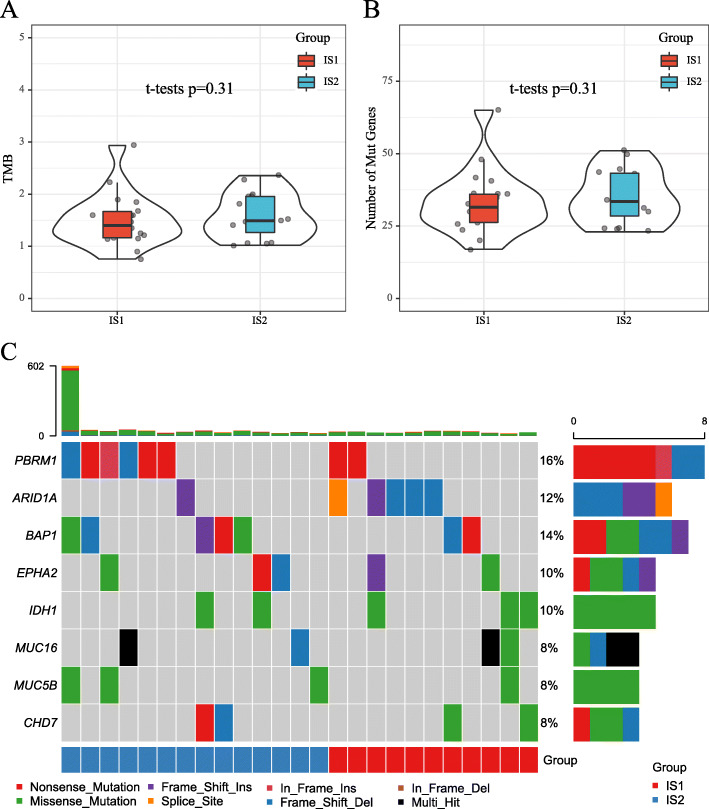
Association of immune subtypes with TMB and mutation. **a**-**b** Tumor mutational burden (TMB) of the two immune subtypes. The mutations per coding region were evaluated in two subtypes (**a**). The number of mutated genes evaluated in the two subtypes (**b**). **c** The landscape of the genomic alteration of 8 representative immune-related genes

Previous studies demonstrated that tumor mutational burden (TMB) and mutation used to quantify the number of tumor antigens is closely related to immunotherapeutic efficacy, including mRNA vaccine [23]. Therefore, the TMB and mutations were assessed using the mutect2-processed mutation dataset in TGCA for the two subtypes. In contrast, no significant difference was observed between the two subtypes in TMB (Fig. 5a) and the number of mutated genes (Fig. 5b). In addition, the landscape of eight immune-related genes with the most frequent genomic alteration was displayed (Fig. 5c). These findings indicate that the amounts of tumor antigens encoded by mutated genes are not significantly different between the two immune subtypes.

### Association of immune subtypes with ICPs and immunogenic cell death modulators

**Fig. 6:**
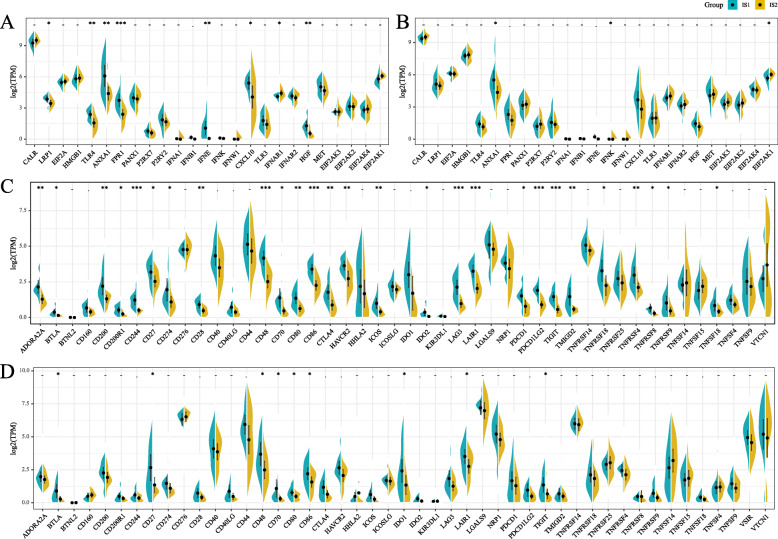
Association of immune subtypes with ICPs and ICD modulators. **a**-**b** Difference in the expression of ICD modulators between the two IS in GEO cohort (**a**) and TCGA cohort (**b**). **c**-**d** Difference in the expression of ICPs between the two IS in GEO cohort (**c**) and TCGA cohort (**d**). **-** *P* ≥ 0.1, **·** *P* < 0.1, * *P* < 0.05, ** *P* < 0.01, *** *P* < 0.001, **** *P* < 0.0001

Previous studies demonstrated that both ICPs (e.g., PD-L1 and TIM-3) and immunogenic cell death (ICD) modulators (e.g., CALR and HMGB1) play critical roles in modulating the host anti-tumor immunity, which could influence the efficacy of mRNA vaccine. Therefore, the differential expression of ICPs and ICD modulators was assessed in the two immune subtypes. A total of 25 ICD modulator-related genes were detected in both GEO and TCGA cohorts. Eight genes were differentially expressed in the two subtypes in the GEO cohort, and IS1 had significantly higher LRP1, TLR4, ANKA1, FPR1, IFNE, CXCL10, and HGF expressions (Fig. 6a). However, the three genes were differentially expressed in the two subtypes in the TCGA cohort, and IS2 had higher IFNK and EIF2AK1 expressions (Fig. 6b). Moreover, 47 ICP-related genes were detected in both GEO and TCGA cohorts. A total of 27 genes were distinctly expressed in the two subtypes in GEO cohort, and IS1 had significant upregulation of ADORA2A, BTLA, CD200, CD200R1, CD244, CD27, CD274, CD28, CD48, CD70, CD80, CD86, CTLA4, ICOS, IDO2, LAG3, LAIR1, PDCD1, PDCD1LG2, TIGIT, TMIGD2, TNFRSF18, TNFRSF4, TNFRSF8, TNFRSF9, TNFSF18 (Fig. 6c). Nine genes were distinctly expressed in the TCGA cohort, and IS1 had significant upregulation of BTLA, CD27, CD48, CD70, CD80, CD86, IDO1, LAIR1, and TIGIT (Fig. 6d). Collectively, the immunotyping can reflect the expression level of ICD modulators and ICPs, thus acting as a biomarker for mRNA vaccine. mRNA vaccine could be less effective to patients with highly expressing ICPs, and more effective to those with upregulation of ICD modulators.

### Association of immune subtypes with CA19–9 and CA125

**Fig. 7:**
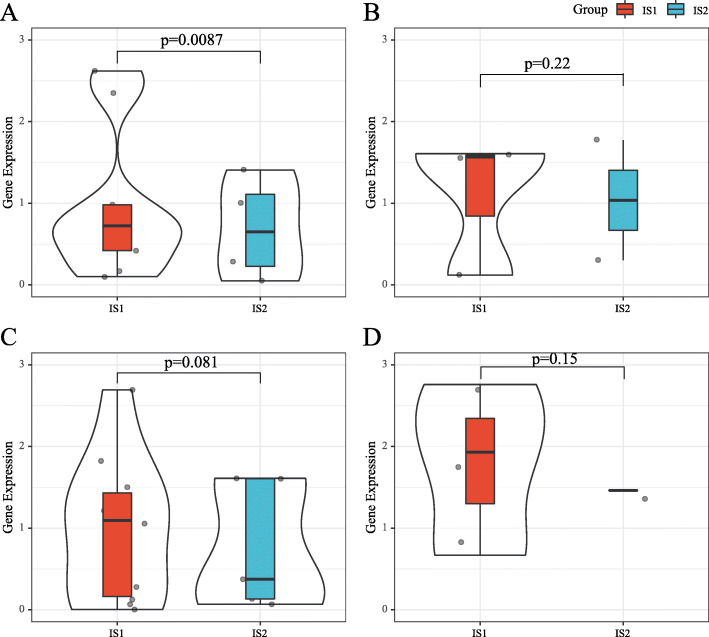
Association of immune subtypes with CA19–9 and CA125. **a-b** Serum CA19–9 and CA125 level, respectively, in the GEO cohort. **c-d** Serum CA19–9 and CA125 level, respectively, in the TCGA cohort

Carbohydrate antigen 19–9 (CA19–9) and carbohydrate antigen 125 (CA125) are the two most commonly used prognostic tumor biomarkers for CHOL and their high value indicates a poor patient outcome. Therefore, the expression levels of CA19–9 and CA125 in each patient in both GEO and TGCA cohorts were analyzed. Serum CA19–9 in IS1 in the GEO cohort were significantly upregulated (Fig. 7a), while there was no significant difference in CA125 between the two subtypes (Fig. 7b). Both CA19–9 and CA125 were not significantly differentially expressed in the two subtypes in the TCGA cohort (Fig. 7c and d), different from the superior prognosis in IS2. Therefore, the prognostic prediction accuracy of immunotype is better than traditional CA19–9 and CA125 in CHOL.

### Cellular and molecular characteristics of the immune subtypes

**Fig. 8:**
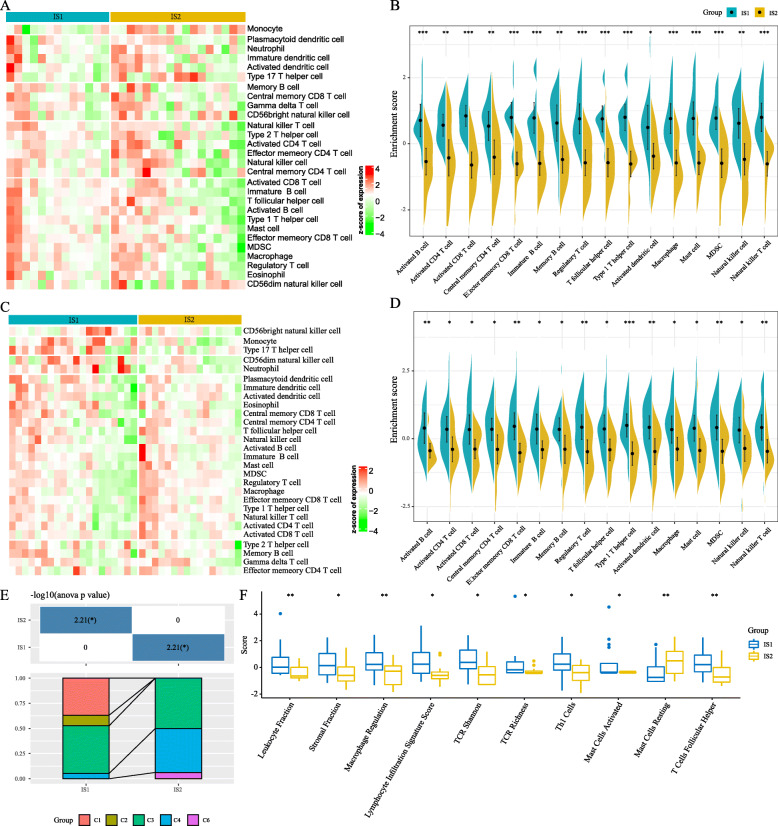
Cellular and molecular characteristics of the immune subtypes. **a**-**b** Heatmap (**a**) and violin plot (**b**) of the relationship between immune subtypes and 28 immune cell subpopulations identified by a previous study in the GEO cohort. **c**-**d** Heatmap (**c**) and violin plot (**d**) of the relationship between immune subtypes and 28 immune cell subpopulations identified by a previous study in the TCGA cohort. **e** Distribution of individual immune categories in the two immune subtypes. **f** Box plot showing the immune-related molecular features in the two immune subtypes. **-** *P* ≥ 0.1, **·***P* < 0.1, * *P* < 0.05, ** *P* < 0.01, *** *P* < 0.001, **** *P* < 0.0001

Since the tumor immune status largely influences the effectiveness of mRNA vaccine, ssGSEA was first used to determine the scores of 28 previously reported immune cells for defining the immune cell components in two immune subtypes [24]. The immune cell components were significantly distinct in the two subtypes (Fig. 8a). IS1 had higher scores in activated B cell, activated CD4^+^ T cell, activated CD8^+^ T cell, central memory CD4^+^ T cell, effector memory CD8^+^ T cell, regulatory T cell, macrophage, and myeloid-derived suppressor cells (MDSC) in the GEO cohort (Fig. 8b), similar to the TCGA cohort (Fig. 8c and d). Therefore, IS1 is an immune “hot” and immunosuppressive phenotype, while IS2 is an immune “cold” phenotype. In a previous study, Thorsson et al. identified six immune categories (C1-C6) based on the immunogenomic analysis of more than 1000 tumor samples among 33 cancer types [21]. These categories were significantly associated with prognosis, genetic, and immune-modulatory alterations in the tumors. Thus, the distribution of the six categories was also investigated in our study. A distinct distribution over IS1 and IS2 was observed, and the individual immune categories substantially varied in their proportion in the two immune subtypes (Fig. 8e). For instance, C1 (wounding healing) and C2 (IFN-r) were mainly clustered into IS1, whereas C4 (immunologically quiet) and C6 (TGF-β dominant) were mainly clustered into IS2. These results suggested that CHOL TIME was extremely different from the TIME of other tumor types, providing a useful and additional complement to previous studies. The relationship between the immune subtypes and 56 previously defined immune-related molecular features was evaluated. The expressions of 10 molecular signatures were significantly different between the two immune subtypes (Fig. 8f). IS1 had higher scores in lymphocyte infiltration, leukocyte fraction, TCR richness, T cells follicular helper, macrophage regulation, mast cell activation, and stromal fraction. IS1 is an immune “hot” and immunosuppressive phenotype, while IS2 is an immune “cold” phenotype, consistent with the cellular signature results. Therefore, immunotyping can be used to mirror CHOL immune status and to distinguish suitable population for mRNA vaccine that could result in immune cell infiltration in patients with immune “cold” IS2.

### The immune landscape of CHOL

**Fig. 9:**
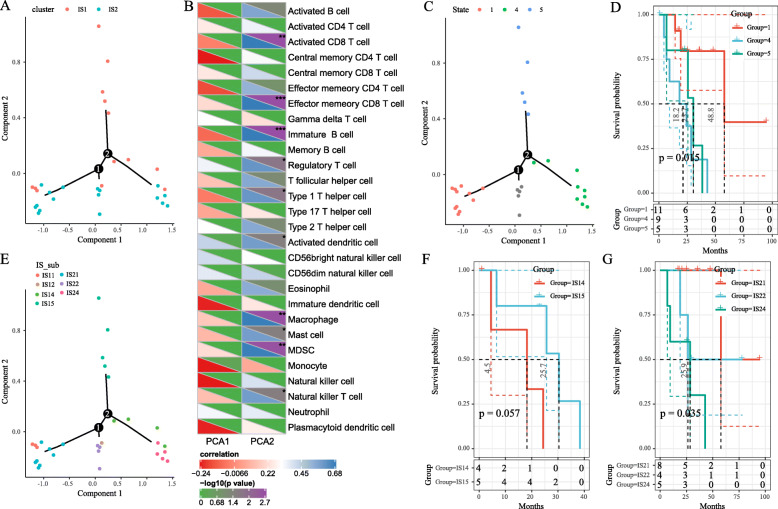
The immune landscape of CHOL. **a** The immune landscape of CHOL. The location of individual patients in the immune landscape with the color corresponding to the immune subtype identified above, which represents the overall characteristics of the TIME. **b** Correlation between PCA1/2 and immune modules. **c**-**d** Patients separated by the immune landscape based on their location (**c**). Separated patients were associated with different prognoses (**d**). **e** Further stratification of IS1 and IS2 based on their location on the immune landscape. **f** Different groups in IS1 associated with different prognoses. **g** Different groups in IS2 associated with different prognoses

The immune-related gene expression profiles were integrated to construct the immune landscape of CHOL to visualize the immune distribution of each patient for mRNA vaccine application (Fig. 9a). The two immune subtypes were oppositely distributed in the immune landscape. The Y-axis was highly correlated with activated CD8 T-cell, effector memory CD8^+^ T cell, immature B-cell, macrophage, and MDSC modules (Fig. 9b). Further prognostic analysis of extremely distributed patients showed that patients in group 1 had better survival probability than group 4 and 5, indicating that immune landscape based on immune subtypes can be used to predict patient prognoses (Fig. 9c and d). IS1 and IS2 were further stratified into distinct subgroups according to the distribution location of the two immune subtypes in the immune landscape (Fig. 9e). Patients with IS1 were divided into IS11, IS12, IS14, and IS15, and those with IS2 were divided into IS21, IS22, and IS24. The prognoses of patients with IS14 were better than those with IS15 (Fig. 9f), while those with IS21 and IS22 had superior prognoses than IS24 (Fig. 9g). Therefore, there is significant intra-cluster heterogeneity within subtypes. Together, these findings suggest that the immune landscape can be used to define the immune components of each CHOL patient and predict prognoses that help in selecting suitable patients for mRNA vaccine.

### Identification of immune gene co-expression modules and immune hub genes of CHOL

**Fig. 10:**
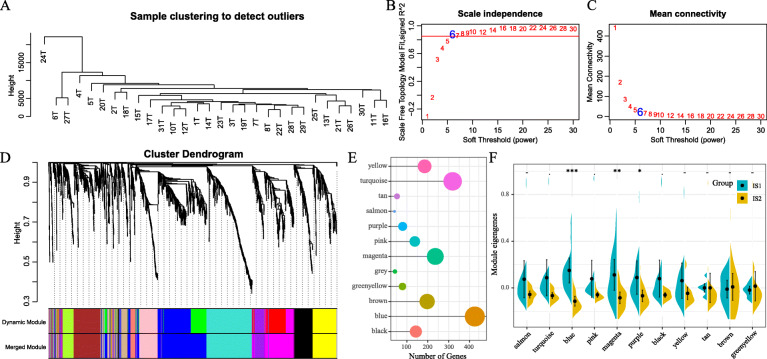
Identification of immune gene co-expression modules. **a**-**d** Gene co-expression network analysis based on the immune-related genes. **e** Dot plot of the co-expression gene modules. **f** Expression of the identified gene modules in the immune subtypes

**Fig. 11:**
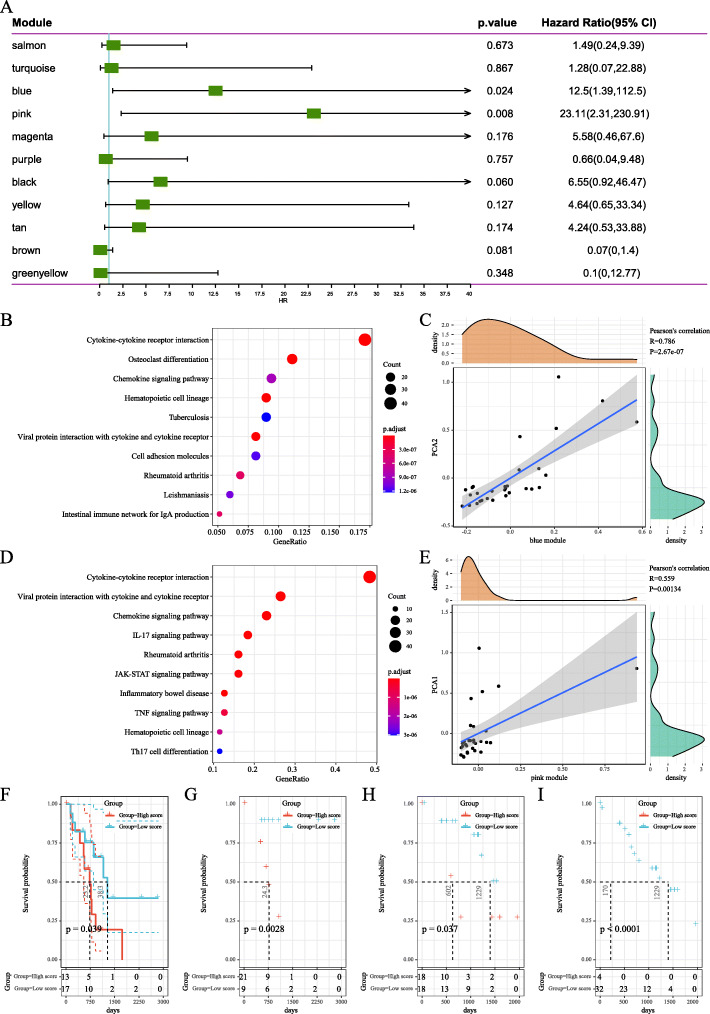
Identification of immune hub genes of CHOL. **a**. Survival analysis of the blue and pink modules. **b** Dot plot of the enrichment results of the biological processes in the blue module. **c** Correlation between gene expression in the blue module and PCA2. **d** Dot plot of the enrichment results of the biological processes in the pink module. **e** Correlation between gene expression in the pink module and PCA1. **f** Gene expression in the two modules contributing to the OS of CHOL patients in the GEO cohort. **g** Gene expression in the two modules contributing to the OS of CHOL patients in the TCGA cohort. **h** Hub gene expression contributing to the OS of CHOL patients in the GEO cohort. **i** Hub gene expression contributing to the OS of CHOL patients in the TCGA cohort

The immune gene co-expression module was used to classify immune-related genes, whose expression significantly influenced the effectiveness of the mRNA vaccine. WGCNA was used to cluster the collected immune-related genes to construct gene modules (Fig. 10a). The soft threshold was set at six in the scale-free network (Fig. 10b and c). The representation matrix was converted to adjacency and next to a topological matrix. The average-linkage hierarchy clustering approach was used with a minimum of 30 genes for each network according to the standard of a hybrid dynamic shear tree. Eigengenes of each module were computed and the close modules were integrated into a new one (height = 0.25, deep split = 3 and min module size = 30) (Fig. 10d). Therefore, 12 gene modules were identified and the genes in the grey module were not clustered with others (Fig. 10e). The module eigengenes in two immune subtypes were then analyzed. The module eigengenes of IS1 were significantly higher in blue, magenta, and purple modules (Fig. 10f). In addition, the prognostic correlation analysis revealed that the expression of genes in the blue and pink modules was significantly associated with the prognosis of CHOL patients (Fig. 11a). Further functional enrichment analysis showed that genes involved in cytokine-cytokine receptor interaction were enriched in the blue module (Fig. 11b), which was significantly positively correlated with component 2 in the immune landscape (Fig. 11c). The pink module enriched with genes in cytokine-cytokine receptor interaction, JAK-STAT signaling pathway, and TNF signaling pathway (Fig. 11d) showed a significantly positive correlation with component 1 in the immune landscape (Fig. 11e). Consistently, patients with low scores of genes clustered into blue (Fig. 11f) and pink (Fig. 11g) modules had prolonged survival compared to those with higher scores in the GEO cohort. Similar trends were observed in the TCGA cohort (Fig. 11h and i). Therefore, patients with highly expressing genes clustered in the blue module are not suitable for mRNA vaccine. In contrast, mRNA vaccine could be effective in patients with the upregulation of genes clustered into the pink module. Finally, 26 immune-related genes with the correlation > 95% to the module eigengenes of blue and pink modules, including CCL11, CCL3L1, CPXM1, CSF3, FAM92B, IFNW1, IL17A, IL1B, IL21, IL22, IL6, IL9, LCE3D, PLA2G4E, PNOC, S100A12, S100A7, S100A7A, S100A9, TNIP3, CCR5, CD53, EVI2B, HCLS1, IL10RA, and PSTPIP1, were immune hub genes. Therefore, the hub genes can act as a biomarker for predicting the prognoses of CHOL patients and for identifying suitable patients for mRNA vaccine.

## Discussion

CHOL is one of the most aggressive malignancies with a heterogeneous molecular profile and limited therapeutic options [1, 2]. The combination of gemcitabine and cisplatin is the first-line option in treating advanced CHOL, with limited clinical benefits and a median OS of just under a year [3]. Although immunotherapy has revolutionized oncology from the therapeutic point of view, its effectiveness in CHOL remains unclear [4, 5]. The mRNA cancer vaccines represent promising novel immunotherapy to treat malignancies because both tumor-specific and non-specific antigens are expressed in cancerous cells. Both of them could be used as mRNA vaccine targets, resulting in the regression of the tumor in both preclinical models and patients [25–27]. Other studies also showed an improved therapeutic efficacy by combining tumor vaccine with immune checkpoint inhibitors or chemotherapy agents [28]. However, the potential effect of the mRNA cancer vaccine is still under exploration in CHOL patients, thus, the clinical benefits are still limited [29].

In this study, the profiles of CHOL somatic mutations and amplified genes were constructed, which revealed a wide range of potent antigen targets that might be considered in CHOL. Since the antigens predicted using the gene alteration profile might not be functionally significant in CHOL, prognostic roles and immune correlations were further analyzed to confirm the clinical relevance of the selected antigens. Three tumor antigens (CD247, FCGR1A, and TRRAP) correlated with superior prognoses and infiltration of antigen-presenting cells in CHOL were identified using narrow-down analysis, thus promising candidates for mRNA vaccine. Although further clinical evaluation is required, the potential of the three tumor antigens to be successful targets for the anti-CHOL mRNA vaccine was consolidated in previous reports. For instance, Xu et al. reported that FCGR1A could be involved in regulation, activation, or induction of immune cells and multiple physiological and pathological processes, thus a potential prognostic biomarker and associated with immune infiltration levels in various cancers, especially CHOL [30].

Since the benefits of therapeutic response and survival of patients subjected to tumor vaccine-based therapy are still limited to a fraction of patients [29, 31], patients with CHOL were stratified based on tumor immune-related gene profile to obtain a guide in the optimal use of tumor vaccine therapy. A distinct gene expression profile and clinical prognosis characterized the two identified immune subtypes. Patients with IS2 had prolonged survival compared with those with IS1, suggesting that immunotype can be a prognostic biomarker for CHOL and its accuracy is better than conventional CA19–9 and CA125. In addition, the immune subtype reflects the expression level of ICD modulators and ICPs. mRNA vaccine could be more effective in patients with upregulation of ICD modulators, while patients with higher ICP expressions were not suitable for mRNA vaccine. Thorsson et al. identified six immune categories among 33 cancer types with distinct immune phenotype and prognosis [32]. The distribution of the six categories in our study was investigated and a different distribution rate of five categories was observed on IS1 and IS2. Other individual immune categories varied substantially in their proportion on the two immune subtypes, except for C3, which was equally distributed in the two isotypes. The majority of C1 (wound healing) and C2 (IFN-g dominant) categories were clustered into IS1 and characterized by an increased immune cell infiltration and relatively better prognosis, while the C4 (lymphocyte depleted) and C6 (TGF-b dominant) categories were clustered into IS2 and associated with an immunologically quiet phenotype and poor prognosis. However, in our study, IS1 tended to have a worse prognosis compared to IS2. These results indicated that CHOL was associated with immune subtypes different from the previously identified categories and our results provided a useful and additional complement in the classification of TIME.

mRNA vaccine is not commonly used in CHOL patients, because of the tumor heterogeneity and its complex tumor immune microenvironment. Unsupervised hierarchical clustering analysis was performed based on a comprehensive set of immune-related genes instead of developing a supervised learning model for patient risk stratification, thus providing new insights into the selection of suitable patients for vaccination. The two subtypes identified in our study showed an extremely distinct TIME. The IS2 displayed an immune desert phenotype characterized by the absence of immune cell infiltration consequently representing a non-inflamed tumor microenvironment. The IS1 showed an opposite immunologic characteristic with an immune-hot phenotype characterized by an increased immune cell infiltration consequently representing an extremely inflamed microenvironment. These two subtypes might represent the different underlying mechanisms regulating tumor immune escape, which should correspond to different treatment strategies. The immune desert phenotype (IS2) might be associated with the lack of tumor antigen and antigen-presenting cells, leading to T cell anergy. A previous study revealed that certain CHOL deregulates the major histocompatibility complex-I (MHC-I) to escape immune surveillance, which is associated with the impairment of immune cell infiltration and poor prognosis [33]. Thus, the use of the mRNA vaccine therapy can induce immune infiltration to reinvigorate the immune system in these patients. The inflamed phenotype (IS1) corresponded to a more complex tumor microenvironment. Previous studies well establish the close relationship between inflammation and CHOL [34, 35]. The inflammatory cytokines, including interleukin-6 (IL-6), tumor necrosis factor-alpha (TNF-α), and transforming growth factor-beta (TGF-β) play critical roles in tumor progression and promote early metastasis [29, 36]. Although IS1 was associated with a high level of immune cell infiltration, the prognosis of IS1 was significantly poorer than the one of IS2. Therefore, the critical factor determining the prognosis might be due to the dominance of the immune-suppressive environment or the stimulatory one. Sylvie et al. analyzed the TME in the intrahepatic CHOL and stratified patients into four subtypes corresponding to the different nature of the TME (lymphoid, myeloid, mesenchymal). The myeloid and mesenchymal dominance resulted in a poor prognosis, while the lymphoid dominance subtype was significantly associated with a good survival [37]. The further graph learning-based dimensionality reduction revealed the intra-cluster heterogeneity in IS1, consistent with the previous study. A fraction of patients (IS1–5) in IS1 showed significantly better survival than others. These patients might be associated with different dominant immune factors that significantly influenced the prognosis of these patients. In these patients, the combination of an mRNA-based cancer vaccine with another immunotherapy or chemotherapy might modulate both the host immune response and tumor microenvironment toward a state more conducive to successful therapy. In addition, patients in IS2–4 had a better prognosis than other groups in IS2. Interestingly, IS2–4 and IS1–5 were closely related to each other having similar positions in the graph, indicating that the two types of patients can have the same treatment strategy. Notably, integrating results of both immune subtypes and the immune landscape of CHOL is important.

Moreover, this study provides important information for mRNA vaccine development for other diseases. For instance, most mRNA vaccines for COVID-19 were developed based on spike (S) protein sequences, whose efficacy could be compromised due to varying escape mutations [38–40]. Furthermore, mRNA vaccine efficacy varies significantly in different groups of recipients [41, 42]. According to this study, identifying the promising specific antigen and patients with corresponding immune subtypes suitable for mRNA vaccine treatment may help improve clinical practice in combatting COVID-19.

## Conclusions

In conclusion, CD247, FCGR1A, and TRRAP are the potential targets of the CHOL mRNA vaccine and could be beneficial for patients with IS2. Thus, this study provides a theoretical foundation for mRNA vaccine against CHOL and defines suitable vaccination patients.

## Data Availability

All data generated and described in this article are available from the corresponding web servers, and are freely available to any scientist wishing to use them for noncommercial purposes, without breaching participant confidentiality. Further information is available from the corresponding author on reasonable request.

## Abbreviations

CHOL: Cholangiocarcinoma
TIME: Tumor immune microenvironment
cBioPortal: cBioPortal for Cancer Genomics
GEPIA: Gene Expression Profiling Interactive Analysis
OS: Overall survival
DFS: Disease-free survival
PAM: Partition around medoids
IGP: In-group-proportion
MSigDB: Molecular Signatures Database
DDRTree: Discriminative dimensionality reduction with trees
CNAs: Copy number alterations
FGA: Fraction of genome altered
PFS: Progression-free survival
IS: Immune subtype
TMB: Tumor mutational burden
ICPs: Immune checkpoints
ICD: Immunogenic cell death
CA19–9: Carbohydrate antigen 19–9
CA125: Carbohydrate antigen 125
dMMR: DNA mismatch repair
MHC-I: Major histocompatibility complex-I
IL-6: Interleukin-6
TNF-α: Tumor necrosis factor-alpha
TGF-β: Transforming growth factor-beta

## References

[1] Rizvi S, Khan SA, Hallemeier CL, Kelley RK, Gores GJ (2018). Cholangiocarcinoma - evolving concepts and therapeutic strategies. Nat Rev Clin Oncol.

[2] Rizvi S, Gores GJ (2013). Pathogenesis, diagnosis, and management of cholangiocarcinoma. Gastroenterology.

[3] Valle J (2010). Cisplatin plus gemcitabine versus gemcitabine for biliary tract cancer. N Engl J Med.

[4] Zappasodi R, Merghoub T, Wolchok JD (2018). Emerging concepts for immune checkpoint blockade-based combination therapies. Cancer Cell.

[5] Ribas A, Wolchok JD (2018). Cancer immunotherapy using checkpoint blockade. Science.

[6] Chen L, Han X (2015). Anti-PD-1/PD-L1 therapy of human cancer: past, present, and future. J Clin Invest.

[7] Sullenger BA, Nair S (2016). From the RNA world to the clinic. Science.

[8] Pardi N, Hogan MJ, Porter FW, Weissman D (2018). mRNA vaccines - a new era in vaccinology. Nat Rev Drug Discov.

[9] Pardi N, Hogan MJ, Weissman D (2020). Recent advances in mRNA vaccine technology. Curr Opin Immunol.

[10] Gu YZ, Zhao X, Song XR (2020). Ex vivo pulsed dendritic cell vaccination against cancer. Acta Pharmacol Sin.

[11] Shahnazari M, Samadi P, Pourjafar M, Jalali A (2020). Therapeutic vaccines for colorectal cancer: the progress and future prospect. Int Immunopharmacol.

[12] Cafri G (2020). mRNA vaccine-induced neoantigen-specific T cell immunity in patients with gastrointestinal cancer. J Clin Invest.

[13] Xu S, Yang K, Li R, Zhang L. mRNA Vaccine Era-Mechanisms, Drug Platform and Clinical Prospection. Int J Mol Sci. 2020;21. 10.3390/ijms21186582. 10.3390/ijms21186582 PMC7554980 32916818

[14] Hainsworth JD (2013). Molecular gene expression profiling to predict the tissue of origin and direct site-specific therapy in patients with carcinoma of unknown primary site: a prospective trial of the Sarah Cannon research institute. J Clin Oncol.

[15] Razumilava N, Gores G (2014). J Cholangiocarcinoma. Lancet.

[16] Raggi C, Invernizzi P, Andersen JB (2015). Impact of microenvironment and stem-like plasticity in cholangiocarcinoma: molecular networks and biological concepts. J Hepatol.

[17] Farshidfar F (2017). Integrative genomic analysis of Cholangiocarcinoma identifies distinct IDH-mutant molecular profiles. Cell Rep.

[18] Cerami E (2012). The cBio cancer genomics portal: an open platform for exploring multidimensional cancer genomics data. Cancer Discov.

[19] Tang Z, Kang B, Li C, Chen T, Zhang Z (2019). GEPIA2: an enhanced web server for large-scale expression profiling and interactive analysis. Nucleic Acids Res.

[20] Li T (2020). TIMER2.0 for analysis of tumor-infiltrating immune cells. Nucleic Acids Res.

[21] Thorsson V (2018). The Immune Landscape of Cancer. Immunity.

[22] Yu G, Wang LG (2012). Han, Y. & he, Q. Y. clusterProfiler: an R package for comparing biological themes among gene clusters. OMICS.

[23] Sha D, et al. Tumor Mutational Burden as a Predictive Biomarker in Solid Tumors. Cancer Discov. 2020. 10.1158/2159-8290.CD-20-0522. 10.1158/2159-8290.CD-20-0522 PMC7710563 33139244

[24] Charoentong P (2017). Pan-cancer Immunogenomic analyses reveal genotype-Immunophenotype relationships and predictors of response to checkpoint blockade. Cell Rep.

[25] Sebastian M (2014). Phase Ib study evaluating a self-adjuvanted mRNA cancer vaccine (RNActive(R)) combined with local radiation as consolidation and maintenance treatment for patients with stage IV non-small cell lung cancer. BMC Cancer.

[26] Li WH, Li YM (2020). Chemical strategies to boost Cancer vaccines. Chem Rev.

[27] Luo W (2020). Novel therapeutic strategies and perspectives for metastatic pancreatic cancer: vaccine therapy is more than just a theory. Cancer Cell Int.

[28] Hailemichael Y (2018). Cancer vaccine formulation dictates synergy with CTLA-4 and PD-L1 checkpoint blockade therapy. J Clin Invest.

[29] Han S, et al. A Perspective on Cell Therapy and Cancer Vaccine in Biliary Tract Cancers (BTCs). Cancers (Basel). 2020;12. 10.3390/cancers12113404. 10.3390/cancers12113404 PMC7698436 33212880

[30] Xu JL, Guo Y (2020). FCGR1A serves as a novel biomarker and correlates with immune infiltration in four Cancer types. Front Mol Biosci.

[31] Guo X, Shen W (2020). Latest evidence on immunotherapy for cholangiocarcinoma. Oncol Lett.

[32] Thorsson V (2019). The immune landscape of Cancer. Immunity.

[33] Goeppert B (2013). Prognostic impact of tumour-infiltrating immune cells on biliary tract cancer. Br J Cancer.

[34] Chapman RW (1999). Risk factors for biliary tract carcinogenesis. Ann Oncol.

[35] Randi G (2009). Epidemiology of biliary tract cancers: an update. Ann Oncol.

[36] Goydos JS (1998). Marked elevation of serum interleukin-6 in patients with cholangiocarcinoma: validation of utility as a clinical marker. Ann Surg.

[37] Job, S. *et al.* Identification of four immune subtypes characterized by distinct composition and functions of tumor microenvironment in intrahepatic cholangiocarcinoma *Hepatology*, doi:10.1002/hep.31092 (2019). 10.1002/hep.31092 PMC7589418 31875970

[38] Liu, Z. *et al.* Landscape analysis of escape variants identifies SARS-CoV-2 spike mutations that attenuate monoclonal and serum antibody neutralization. *bioRxiv*, doi:10.1101/2020.11.06.372037 (2020). 10.1016/j.chom.2021.01.014 PMC7839837 33535027

[39] Lu S (2021). The immunodominant and neutralization linear epitopes for SARS-CoV-2. Cell Rep.

[40] Zhang L (2020). SARS-CoV-2 spike-protein D614G mutation increases virion spike density and infectivity. Nat Commun.

[41] Khani E, Khiali S, Entezari-Maleki T. Potential COVID-19 therapeutic agents and vaccines: an evidence-based review. J Clin Pharmacol. 2021. 10.1002/jcph.1822. 10.1002/jcph.1822 PMC8014753 33511638

[42] Kim JH, Marks F, Clemens JD. Looking beyond COVID-19 vaccine phase 3 trials. Nat Med. 2021. 10.1038/s41591-021-01230-y. 10.1038/s41591-021-01230-y 33469205

